# Modulation of Intestinal Corticotropin-Releasing Hormone Signaling by the Herbal Preparation STW 5-II: Possible Mechanisms for Irritable Bowel Syndrome Management

**DOI:** 10.3390/ph15091121

**Published:** 2022-09-08

**Authors:** Mohamed Elbadawi, Ramy M. Ammar, Sabine Rabini, Sabine M. Klauck, Thomas Efferth

**Affiliations:** 1 Department of Pharmaceutical Biology, Institute of Pharmaceutical and Biomedical Sciences, Johannes Gutenberg University-Mainz, 55128 Mainz, Germany; 2 Medical Affairs, Bayer Consumer Health, 64295 Darmstadt, Germany; 3 Division of Cancer Genome Research, German Cancer Research Center (DKFZ), German Cancer Consortium (DKTK), National Center for Tumor Diseases (NCT), 69120 Heidelberg, Germany

**Keywords:** irritable bowel syndrome, mouse intestinal organoids, corticotropin-releasing factor, gut-brain axis, tight junctions, intestinal inflammation, proinflammatory cytokines, stress

## Abstract

Corticotropin-releasing factor (CRF) mediates stress responses and alters the gut-brain axis, contributing to the pathogenesis of irritable bowel syndrome (IBS), which is recognized by abdominal pain accompanied by bowel habit disturbance. STW 5-II, a mixture of six herbal extracts, is clinically effective in functional dyspepsia and IBS. Here we aimed to establish an organoid-based stress-induced IBS-like model to investigate the mechanisms of action of STW 5-II. STW 5-II (10, 20, and 30 g/mL) was applied to intestinal organoids for 24 h before being treated with CRF (100 nM) for 48 h. The effects of STW 5-II on CRF signaling were investigated using several in vitro and in silico approaches. STW 5-II activities were further explored by in silico PyRx screening followed by molecular docking of the main 52 identified compounds in STW 5-II with both CRF receptors CRFR1 and CRFR2. CRF exposure stimulated inflammation and increased proinflammatory mediators, while STW 5-II dose-dependently counteracted these effects. STW 5-II inhibited CRF-induced claudin-2 overexpression and serotonin release. Docking of the STW 5-II constituents oleanolic acid and licorice saponin G2 to CRFR1 and CRFR2, respectively, showed a good affinity. These multi-target activities support and elucidate the clinically proven efficacy of STW 5-II in disorders of gut-brain interaction.

## 1. Introduction

Irritable bowel syndrome (IBS) is a functional bowel disorder associated with abdominal discomfort and alteration of bowel habits, with no existing organic alterations. IBS is categorized into four subtypes: IBS with predominant constipation (IBS-C), IBS with predominant diarrhea (IBS-D), IBS with mixed bowel habits (IBS-M), and unclassified IBS (IBS-U) [1]. IBS is a multifactorial disorder with incompletely understood pathophysiology. Based on recent studies that propose the involvement of the gut-brain axis on the generation of these disorders, IBS and other functional gastrointestinal (GI) disorders have been reclassified as disorders of gut-brain interactions (DGBI) characterized by symptoms that are attributed to the following pathologic features: visceral hypersensitivity, dysmotility, altered mucosal function, local intestinal inflammation, dysbiosis, and altered signaling of the central nervous system (CNS) [2].

The gut-brain axis (GBA) is a bidirectional communication system with multiple players, including sensory and motor nerves (neural components), the endocrine system, gastrointestinal components, and the immune system. GBA dysregulation substantially contributes to the development of IBS-related conditions, such as dysmotility, visceral hypersensitivity, abdominal distention, and pain [3,4]. Chronic stress can increase susceptibility to developing IBS and contributes to its symptoms exacerbation [5]. The body’s response to a physical or psychological stimulus that affects the organism’s homeostasis is referred to as stress. The biological effects of stress are mediated by neuro-immune mechanisms, including corticotropin-releasing factor (CRF)/hypothalamic–pituitary–adrenal axis (HPA) pathways and the sympathetic nervous system. Stress-mediated stimulation or interaction of the CRF and HPA axis systems in animal models has been linked to visceral hypersensitivity, a key feature of IBS pathogenesis. Compared to healthy subjects, IBS patients exhibit greater reactivity to stress, manifested by a dysregulated HPA axis response [6]. Consequently, stress has widespread effects on gastrointestinal physiology, including alteration in intestinal motility, abnormal mucosal transport, and disrupted intestinal barrier function, which may affect paracellular permeability and visceral sensitivity.

CRF is a 41-amino acid protein that was initially discovered in the bovine hypothalamus. Later research identified CRF-related proteins, notably urocortin 1, urocortin 2, and urocortin 3, that have sequence homologies and functional features with CRF [7]. To date, two CRF receptors have been cloned and their downstream signaling pathways described. CRF and urocortins exert their actions via the activation of these receptors, and show markedly different affinities towards the CRF1 and CRF2 receptors [8]. Several studies have examined the expression of CRF receptors in the intestine, demonstrating that CRF receptors are differentially expressed in the colon, ileum, and other intestinal parts [9].

CRF plays an important role in the pathogenicity of several GI disorders, including IBS. Pharmacological investigations reveal that central CRF injection induces various IBS-related symptoms, including visceral hypersensitivity and discomfort, abdominal distention, watery stool, and increased intestinal permeability [7]. Moreover, CRF induces the expression of several proinflammatory cytokines such as TNFα, IL-1β, and IL-6—which, in turn, lead to intestinal permeability related to alteration of tight junctions. Furthermore, CRF induces the activation of TLR4 and NF-κB, which is a key regulator of intestinal inflammation [10,11].

STW 5-II is a new herbal formula, combining the extracts of six medicinal plants: *Iberis amara* L., *Glycyrrhiza glabra* L., *Chamomilla recutita* R., *Menthae piperitae* L., *Melissa officinalis* L., and *Carum carvi* L. The clinical efficacy of STW 5-II in IBS and functional dyspepsia has been evidenced in several randomized prospective clinical trials and numerous preclinical mechanistic studies [12,13,14]. We recently reported its anti-inflammatory and tight junction-protective activities in an ex vivo IBS-like model [15].

The current study aims to evaluate the impact of STW 5-II in a stress-mediated IBS-like model by analyzing its effects on modulating CRF receptor signaling and consequent intestinal inflammation, using the mouse intestinal organoids model.

## 2. Results

### 2.1. PyRx Screening and Docking

PyRx screening revealed that the 52 compounds of STW 5-II bind to the CRF receptors with considerable affinity. Oleanolic showed the highest binding affinity to CRFR1, while licorice saponin G2 exhibited the highest binding affinity to CRFR2. Table 1 and Table 2 present the top 10 ligands with the highest binding affinity for both receptors.

**Figure 1:**
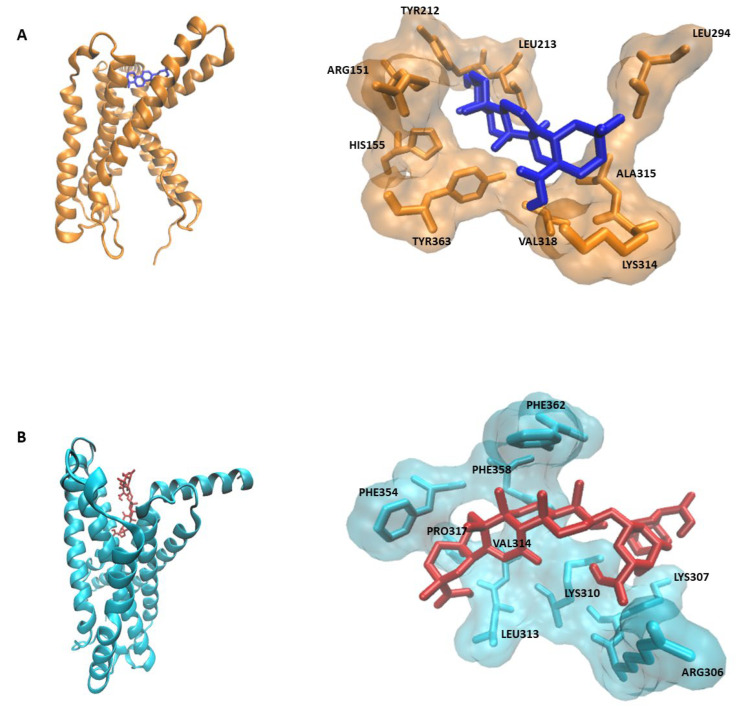
Docking results. (**A**) Binding interactions of oleanolic acid with CRFR1. (**B**) Binding interactions of licorice saponin G2 with CRFR2.

Molecular docking analyses revealed that oleanolic acid bound to CRFR1 with a binding energy of −8.6 ± 0.01 kcal/mol and a pKi of 496.6 ± 9.1 nM. In contrast, licorice saponin G2 interacted with a binding energy of −10.07 ± ≤0.001 kcal/mol and a pKi of 41.36 ± ≤0.001. The docking results are shown in Table 3 and Figure 1.

### 2.2. Microarray Analyses

**Figure 2:**
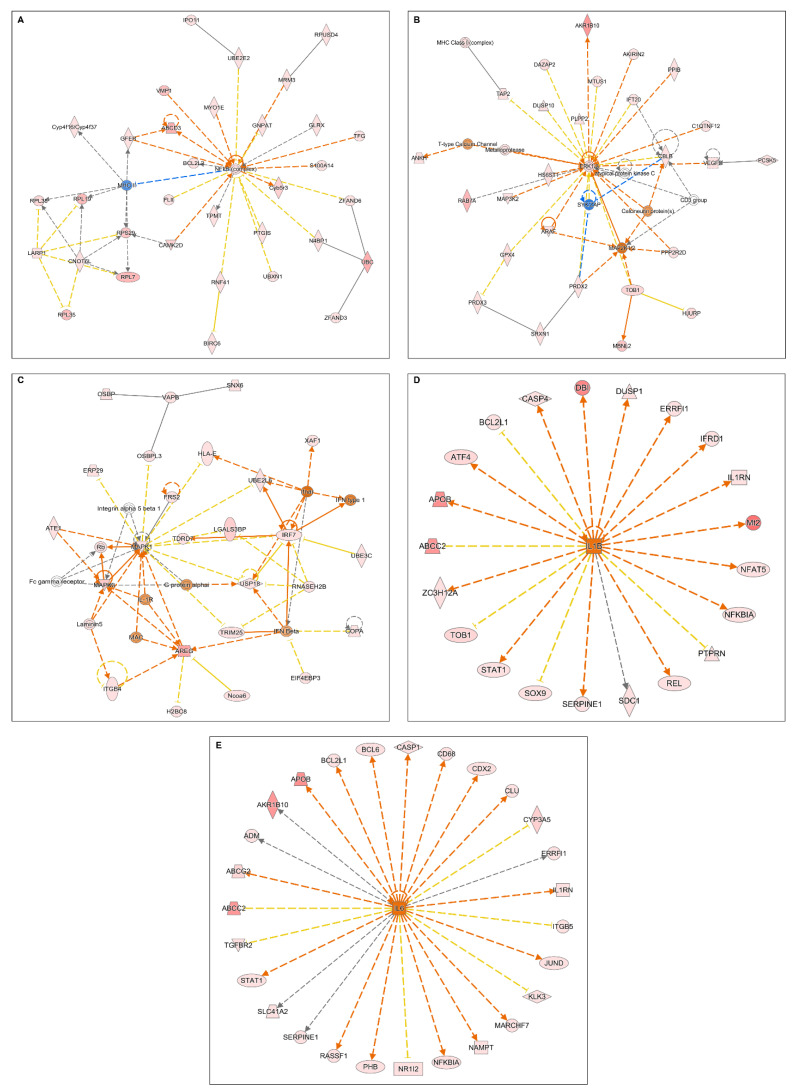
Microarray analyses. (**A**–**C**) Altered cellular networks and mediators after treatment with CRF. (**D**,**E**) Upregulation of upstream regulators (IL-1β and IL-6) after treatment with CRF.

Microarray analyses were performed to highlight the mechanism of inflammation induction by CRF, and the potential modulatory effects of STW 5-II on CRF signaling. Treatment with CRF alone led to the activation of many inflammatory mediators and transcription factors, including NF-κB complex, cytokine receptors, and proinflammatory cytokines, such as IL-6 and IL-1β. Moreover, CRF stimulated the upregulation of MAP kinases and ERK1/2, which are downstream regulators of CRF receptor signaling (Figure 2A–E).

**Figure 3:**
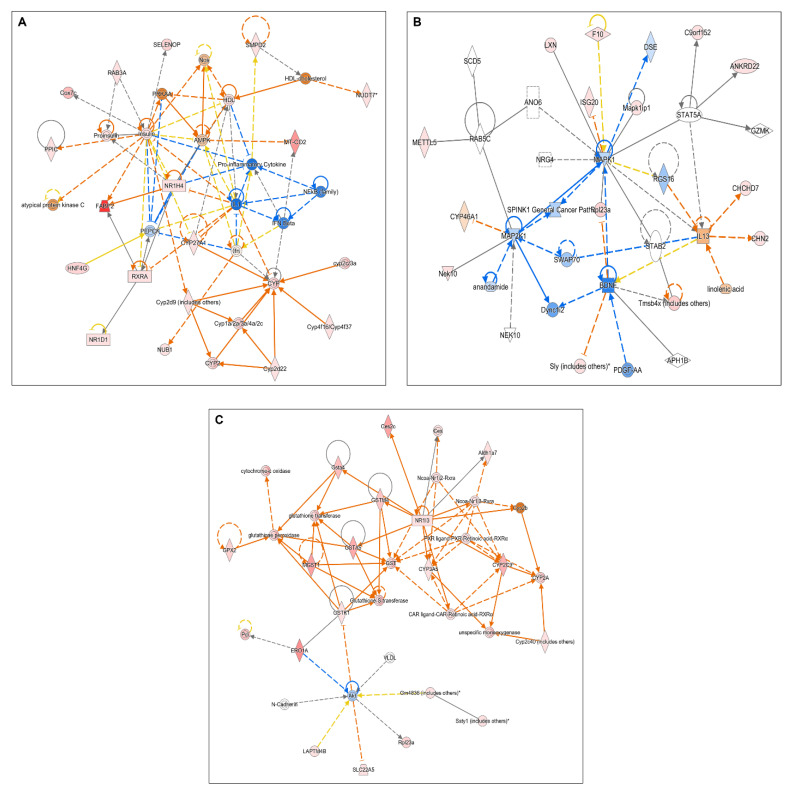
Microarray analyses. (**A**–**C**) Altered cellular networks and mediators after simultaneous treatment with CRF and STW 5-II. The addition of STW 5-II downregulates some proinflammatory mediators such as IL-1, IFN beta, and NF-κB.

In contrast, the simultaneous treatment of organoids with CRF and STW 5-II led to the downregulation of NF-κB, the proinflammatory cytokine IL-1, IFN-β, and other inflammatory mediators. Furthermore, MAP kinases were downregulated, indicating that STW 5-II modulated the downstream regulators of CRF receptor signaling (Figure 3A–C).

### 2.3. Quantitative Real-Time PCR (qPCR)

**Figure 4:**
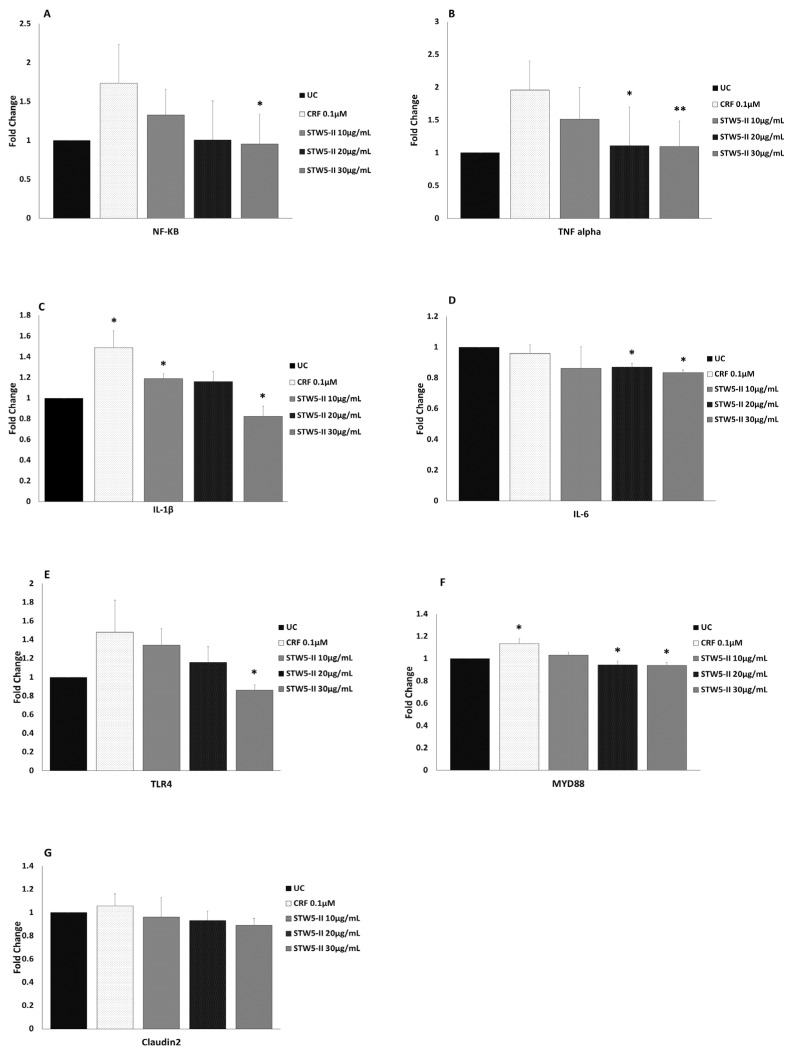
(**A**–**D**) Expression of NF-κB and some proinflammatory cytokines (TNF alpha, IL-1β, and IL-6) after treatment with CRF (100 nM) for 48 h alone and with STW 5-II. CRF treatment increased the expressions of NF-κB, TNFα, and IL-1β, and slightly decreased IL-6, while pretreatment with increasing concentrations of STW 5-II (10, 20, and 30 µg/mL) downregulated the expressions of these mediators. (**E**,**F**) Expression of TLR4 and its downstream mediator MYD88 after treatment with CRF alone and with STW 5-II. The expressions of these mediators showed a similar pattern to that of cytokines. (**G**) Claudin-2 expression in organoids. Treatment with CRF (100 nM) slightly increased Claudin-2 expression, while pretreatment with increasing concentrations of STW 5-II (10, 20, and 30 µg/mL) downregulated Claudin-2 expression. Statistical significance relative to control or CRF is indicated at a level of * *p* < 0.05 or ** *p* < 0.01.

These multiple activities of STW 5-II were confirmed by using qPCR to study the effects of CRF and STW 5-II on the expressions of inflammatory mediators, cytokines, and tight junction proteins. Treatment with CRF (100 nM) for 48 h increased NF-κB expression, while pretreatment for 24 h with increasing concentrations of STW 5-II (10, 20, and 30 µg/mL) decreased NF-κB expression in a dose-dependent manner. This effect of STW 5-II was significant at a concentration of 30 µg/mL (*p* = 0.022) (Figure 4A).

A similar pattern was observed for proinflammatory cytokines. CRF alone led to upregulation of *TNFα*, and this effect was dose-dependently abrogated by pretreatment with STW 5-II. This STW 5-II-induced activity was significant at the higher concentrations of 20 and 30 µg/mL (*p* = 0.011 and *p* = 0.006, respectively) (Figure 4B). Interestingly, CRF treatment significantly increased *IL-1β* expression (*p* = 0.018), while STW 5-II dose-dependently reduced its expression. This activity was considered significant at STW 5-II concentrations of 10 and 30 µg/mL (*p* = 0.025 and *p* = 0.024, respectively) (Figure 4C).

Surprisingly, treatment with CRF (100 nM) resulted in a slight decrease of *IL-6* expression. This effect was augmented by additional treatment with STW 5-II, yielding significant reductions of *IL-6* expression at STW 5-II concentrations of 20 µg/mL and 30 µg/mL (*p* = 0.033 and *p* = 0.025, respectively) (Figure 4D). CRF increased *TLR4* expression, while STW 5-II reversed this effect and yielded significant inhibition at a concentration of 30 µg/mL (*p* = 0.035) (Figure 4E). Moreover, CRF significantly upregulated *MYD88* (*p* = 0.031), an important downstream signaling mediator of *TLR4*. STW5-II inhibited CRF-mediated *MYD88* upregulation, showing more significant effects at concentrations of 20 µg/mL and 30 µg/mL (*p* = 0.018 and *p* = 0.016, respectively) (Figure 4F). Apart from inflammatory mediators, CRF slightly increased expression of the tight junction protein *Claudin-2*, and this effect was reversed by pretreatment with STW 5-II (Figure 4G).

Overall, the qPCR results were strongly correlated to the microarray results (R = 0.907).

### 2.4. Whole-Mount Immunofluorescence Staining

**Figure 5:**
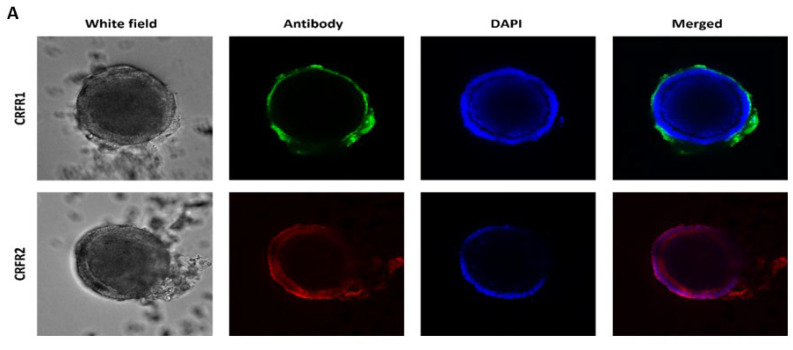

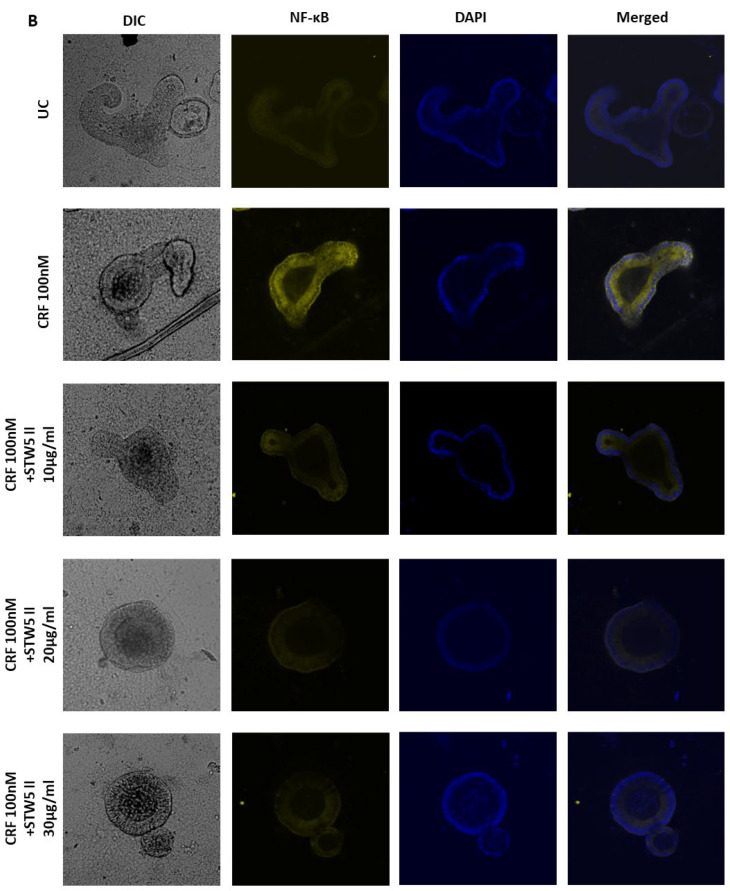
(**A**) Expression of CRF receptors within mouse intestinal organoids. CRF receptor 1 (CRFR1) is localized in the margins of the organoids, while CRF receptor 2 (CRFR2) is spread all over the organoid. (**B**) Expression of NF-κB in intestinal organoids after treatment with CRF (100 nM) for 48 h alone and with STW 5-II. CRF treatment increased NF-κB expression, while pretreatment with increasing concentrations of STW 5-II (10, 20, and 30 µg/mL) downregulated NF-κB expression.

Whole-mount immunofluorescence staining of CRF receptors showed that both CRFR1 and CRFR2 were expressed in mouse intestinal organoids, with different expression patterns and levels. Immunofluorescence staining revealed CRFR1 expression in the organoid margins, while CRFR2 expression was spread all over the organoids (Figure 5A).

NF-κB staining revealed a pattern similar to that observed in qPCR analyses. Treatment of organoids with 100 nM CRF increased the NF-κB expression, while pretreatment with STW 5-II rescued from this effect. STW 5-II at concentrations of 10 and 20 µg/mL led to dose-dependent NF-κB downregulation, while a dose of 30 µg/mL yielded an effect like that produced by 20 µg/mL STW 5-II (Figure 5B).

### 2.5. Detection of Serotonin by ELISA

**Figure 6:**
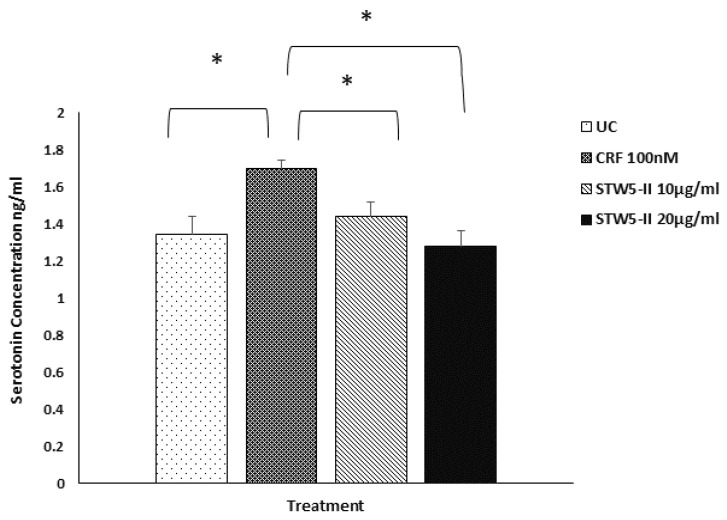
Serotonin detection by ELISA in mouse intestinal organoids after treatment with CRF (100 nM) for 48 h alone and with STW 5-II. CRF treatment increased the level of serotonin in the culture medium, while pretreatment with increasing concentrations of STW 5-II (10 and 20 µg/mL) downregulated serotonin expression. * Denotes statistical significance relative to CRF at a level of *p* < 0.05.

ELISA was performed to detect the level of secreted serotonin in the culture medium after treatment with CRF alone and with CRF plus STW 5-II. Treatment with CRF significantly increased the concentration of serotonin (*p* = 0.011), while additional treatment with STW 5-II (10 or 20 µg/mL) significantly reduced the serotonin concentration (*p* = 0.043 and *p* = 0.027, respectively) (Figure 6).

## 3. Discussion

IBS is among the most prevalent disorders of gut-brain interaction. Since several factors contribute to its pathogenesis, a multi-target therapeutic approach should be followed to manage its complications and underlying pathogenic causes. The multi-herbal preparation STW 5-II is a promising solution that appears to act efficiently and synergistically in targeting several pathological mechanisms, including inflammation, alteration of intestinal permeability, and stress-related signaling pathways. Previous studies have applied several in vitro and in vivo models—including IBS models, inflammatory bowel disorder (IBD) mouse models, and stress models—to study CRF signaling and stress pathways in the brain and intestine [16,17]. Nevertheless, to the best of our knowledge, this is the first study that utilizes an ex vivo 3D intestinal organoid model to investigate CRF receptor expression and related signaling pathways, as well as CRF-induced inflammation.

Intestinal organoids are 3D models generated from stem cells, which exhibit structural and functional similarities to the normal intestine. To date, they are the best in vitro/ex vivo models for studying intestinal physiology and diseases, since they contain most of the underlying cells types [18].

In general, CRF contributes to IBS pathogenesis through the induction of several mechanisms, such as visceral hypersensitivity, intestinal inflammation, abdominal distention, and altered paracellular permeability [19]. There have been contradictory reports regarding the role of each CRF receptor. While most studies support the proinflammatory role of CRFR1, and the anti-inflammatory role of CRFR2, a few studies describe a potential proinflammatory role of CRFR2 [8,20]. Nevertheless, evidence from pharmacological studies, using nonselective and selective receptor antagonists, shows that CRFR1 is more involved in inflammation and mucosal barrier dysfunction [10]. Our present docking analyses showed that some potential components of STW 5-II bound to CRF receptors with high affinity, which may explain the STW 5-II bioactivities.

NF-κB is a key player involved in GI tract physiology and pathophysiology which contributes to the development of functional disorders, such as IBS [21]. Previous evidence shows that CRF induces the expression of intestinal NF-κB—thereby aggravating intestinal inflammation, causing cytokine release, and leading to alterations of intestinal permeability [22]. Interestingly, a quantitative real-time PCR revealed that STW 5-II dose-dependently attenuated the CRF-mediated upregulation of NF-κB. This finding was confirmed by whole-mount immunofluorescence staining of the organoids.

Proinflammatory cytokines, such as IL-6, IL-1β, and TNFα, are involved in intestinal immune regulation, as well as in pathogenic conditions, such as IBS and IBD, as they can induce intestinal mucosal damage, altered permeability, and visceral hypersensitivity [23]. Moreover, cytokine secretion can be stimulated by stress and alteration of the gut-brain axis. It has also been demonstrated that CRF induces the release of IL-6, IL-1β, and TNFα, which may lead to further intestinal complications [24]. In the current study, we observed a similar pattern of CRF-mediated cytokine stimulation. CRF at a concentration of 100 nM induced the expressions of IL-6, IL-1β, and TNFα. Remarkably, STW 5-II effectively abrogated these effects and dose-dependently reduced the expressions of IL-6, IL-1β, and TNFα.

Previous studies have demonstrated a link between activation of CRF signaling and the toll-like receptor 4 (TLR4) system. CRF, via CRFR1, increases colonic permeability and distension through activation of TLR4 and IL-1. Not surprisingly, this effect is abolished by TLR4 and IL-1 antagonists [11]. It is also well established that this activity involves Myd88, the adapter protein of TLR4 [25]. Remarkably, our results confirmed the link between CRF and induction of *TLR4* and *Myd88* expression, with a more notable effect on Myd88 expression. Moreover, STW 5-II showed inhibitory activity by reducing *TLR4* and *Myd88* expression.

Intestinal dysfunction and altered intestinal permeability are substantially involved in IBS pathology, and associated with symptoms such as visceral pain and hypersensitivity and altered bowel habits. This may be mediated through cytokine secretion and alterations of the expressions of tight junction proteins [26,27]. Notably, CRF alters the expressions of several tight junction proteins, including claudin-2, ZO-1, claudin-1, and occludin. In contrast to other tight junction proteins, claudin-2 compromises the barrier functions and increases intestinal permeability [28,29]. Consistent with the literature, we found that CRF treatment led to upregulated *claudin-2* expression. Moreover, STW 5-II effectively reversed this effect and reduced the expression of *claudin-2*.

The neurotransmitter Serotonin (5-hydroxytryptamine, 5-HT) is expressed in several organs and plays an important physiological role [30,31]. Approximately 95% of serotonin in the body resides in the gut, and is secreted by enterochromaffin (EC) cells, as well as serotonergic neurons [32]. Serotonin plays an essential role in regulating GI motility and secretions, modulating sensory information, and coordinating reflexes [33]. Several studies demonstrate that serotonin signaling alteration plays a role in the pathogenicity of IBS, chronic constipation, or diarrhea. Alterations in mucosal serotonin concentration and EC cell functions have been associated with GI dysmotility, visceral hypersensitivity and secretomotor abnormalities in patients with both IBS-C and IBS-D [34,35]. Here we found elevated serotonin levels in the organoids upon CRF treatment which decreased when STW 5-II was applied in parallel.

## 4. Materials and Methods

### 4.1. Materials

STW 5-II was provided in the form of dry lyophilizate by Bayer Consumer Health (Steigerwald Arzneimittelwerk GmbH, Darmstadt, Germany). We used the same batch that had been previously analyzed by high performance liquid chromatography/mass spectrometry (HPLC/MS) for its phytochemical composition [15]. This batch complied with the quality specs of STW 5-II commercial batches. Distilled water was used to prepare the stock solution as well as dilutions of STW 5-II. The mouse intestinal organoids were purchased from STEMCELL Technologies (Cologne, Germany).

### 4.2. Mouse Intestinal Organoid Culture

Mouse intestinal organoids were cultured in a mixture of Matrigel (Corning, New York, NY, USA) and organoid growth medium (Intesticult; STEMCELL Technologies). Briefly, organoid pellets were suspended in a mixture of Matrigel and Intesticult at a ratio of 1:1. The organoids were then seeded in 6-well plates, with 4–5 Matrigel domes per well. The plates were incubated at 37 °C for 20 min to allow Matrigel solidification, and then 2 mL Intesticult was added to each well. The culture was maintained for 7–14 days before the next passage.

### 4.3. Stress-Induced IBS-Like Model

To simulate IBS-associated conditions, CRF was applied to induce inflammation and intestinal barrier disruption. Based on previous studies [12,15], the organoids were pre-treated with different doses of STW 5-II (10, 20, 30 μg/mL) and incubated for 24 h. Next, stress-induced inflammation was modeled by adding 100 nM murine CRF (Cloud-Clone Corp., Houston, TX, USA), followed by incubation for 48 h before subsequent experiments.

### 4.4. PyRx Screening

For 52 compounds identified in STW5-II using LC-MS [15], the 3D structures were drawn using Chemsketch V5 (https://www.acdlabs.com/products/chemsketch/). PyRx (https://pyrx.sourceforge.io) was used for preliminary screening and prediction of activity against the corticotropin-releasing factor receptors CRFR1 and CRFR2.

### 4.5. Microarray Analyses

Microarray analyses were performed to study the detailed molecular mechanisms of STW 5-II and its interaction with CRF signaling. Briefly, STW 5-II was applied to mouse intestinal organoids for 24 h, and then treated with 100 nM murine CRF for 48 h. Next, total RNA was extracted using the InviTrap^®^ Spin Universal RNA Mini Kit (Invitek Molecular, Berlin, Germany). Subsequently, cDNA was synthesized and labeled at the Genomics and Proteomics Core Facility at the German Cancer Research Center (DKFZ, Heidelberg, Germany), and hybridization was conducted on Affymetrix GeneChips^®^ with mouse Clariom S assays (Affymetrix, Santa Clara, CA, USA). The raw microarray data were processed utilizing Chipster software (http://chipster.csc.fi/) to sort out the genes based on variable expression and statistical significance using an empirical Bayes *t*-test. The data from Chipster were analyzed by Ingenuity Pathway Analysis software (IPA; Ingenuity Systems, Qiagen, Redwood City, CA, USA) to uncover the molecular networks and pathways altered by CRF and STW 5-II.

### 4.6. Molecular Docking

Molecular docking was performed for the compounds with the highest PyRx binding energies. Homology models of mouse CRFR1 and CRFR2 were generated utilizing the online tool SWISS-MODEL (https://swissmodel.expasy.org/) (accessed on 15 March 2021). These models were subjected to further preparation and structural refinement using Autodock Tools-1.5.6rc3 [36,37] to resolve any structure-associated problems. Defined Molecular docking was then performed using AutoDock 4. The results were expressed in terms of binding energies, inhibition constant (Ki) values, and amino acid–ligand interactions.

### 4.7. Quantitative Real-Time Reverse Transcription PCR

Quantitative real-time PCR was conducted to study the expression of several important genes. Briefly, 1 µg of extracted RNA was converted to the complementary DNA using the LunaScript^®^ RT SuperMix Kit cDNA Synthesis Kit (New England Bio Labs, Darmstadt, Germany). Next, amplification of seven genes was performed using Eva green master mix (5× Hot Start Taq EvaGreen^®^ qPCR Mix (no ROX); Axon Labortechnik, Kaiserslautern, Germany) as described in the manufacturer’s protocol.

All PCR primers were designed using the NCBI/Primer-BLAST online tool, and their quality was checked with Eurofins MWG Operon (Ebersberg, Germany). Table 4 lists the sequences of primers for selected genes and *GAPDH* (as a control). Using 384-well plates, real-time PCR was operated for 40 cycles on CFX384™ (Bio-Rad, Munich, Germany). The run conditions included a denaturation step at 95 °C for 15 s, succeeded by gradient annealing temperatures of 62–47 °C for 30 s, and lastly a 1-min elongation step at 72 °C. CFX Manager Software (version 3.1; Bio-Rad) was used to define the Cq values. The comparative Cq (2−ΔΔCq) approach was used to calculate the fold-change in gene expression [38].

### 4.8. Whole-Mount Immunofluorescent Staining

Whole-mount immunofluorescent staining was performed to detect the expression of CRF receptors in mouse intestinal organoids and to study the effect of STW 5-II on NF-κB expression. Mouse intestinal organoids were cultured in an 8-well slide chamber (Ibidi, Germany). After their maturation for two to three days, the multiherbal preparation STW 5-II (10, 20, 30 µg/mL) was added. Following a 24-h incubation, the organoids were treated for 48 h with murine CRF (100 nM). The organoids were then rinsed in washing buffer (0.1% Triton X 100 in PBS) and fixed for 30 min at room temperature with 4% formaldehyde. Subsequently, the organoids were washed for 10 min with washing buffer three times, then permeabilized with a permeabilization reagent (0.5% Triton X in PBS) for 1 h followed by washing. For optimum visualization, the organoids were cleared with tissue clearing reagent CUBIC-L (TCI chemicals, Germany) for 1 h and then washed extensively. To prevent non-specific protein binding, organoids were incubated with blocking buffer (4% BSA, 10% FBS in PBS) for 1-h. Subsequently, 350 µL of the primary antibody against NF-κB (diluted 1:900 in blocking buffer; Cell signaling) was added and incubated in a humified chamber at 4 °C overnight. The primary antibody was removed the next day, and the slides were thoroughly washed for 10 min three times. For optimal clearing, a second clearing step was performed using CUBIC-R (TCI chemicals, Eschborn, Germany) for 1 h at room temperature. Following three washes, an anti-rabbit secondary antibody (Alexa Fluor^®^ 488 Conjugate; diluted 1:750; Cell signaling) was added for a 2-h incubation in a humified chamber at room temperature in the dark.

The same steps were followed for the detection of CRF receptors, except for treatment with either STW 5-II or CRF. For this experiment, the primary antibodies were CRHR1 and CRHR2 rabbit polyclonal antibodies (2 µg/mL) (Invitrogen, Waltham, MA, USA).

Nuclear staining was done for 5 min with 4′,6-diamidino-2-phenylindole DAPI (1 µg/mL) and then washed three times. Lastly, the organoids were covered with mounting medium (Ibidi, Gräfelfing, Germany) and imaged with a Visitron spinning disk confocal microscope at the Institute of Molecular Biology’s Microscopy & Histology Core Facility (IMB, Mainz, Germany).

### 4.9. Detection of Serotonin by ELISA

Serotonin in the cell culture medium was assessed using the Serotonin ELISA kit ADI-900-175 (Enzo life sciences, New York, NY, USA). Mouse intestinal organoids were treated with STW 5-II and CRF, as previously described. Before starting the assay, an assay buffer was used to dilute the samples [untreated control (UC), CRF, and STW 5-II 10–20 µg/mL] (1:4), serotonin antibody, serotonin standard, and serotonin conjugate.

Two-fold serial dilutions of serotonin (from 500–0.49 ng/mL) were prepared, and 100 µL of each concentration was added to 96-well plates in duplicate. Additionally, 100 µL of each sample was added to the appropriate wells. For the non-specific binding (NSB) wells and Bo (0 ng/mL), we added 150 µL and 100 µL of assay buffer, respectively. Next, 50 µL serotonin conjugate was added to each well except for the total activity (TA) wells. Consequently, 50 µL serotonin antibody was added to each well (excluding the blank, TA, and NSB wells), and the plate was sealed and incubated at room temperature for 2 h on a plate shaker. The plate was then emptied, washed three times with wash buffer, and dried over a paper towel to remove any residual wash buffer. Next, 5 µL of the conjugate (diluted 1:20) was added to the TA wells, followed by 200 µL of the substrate solution into each well. The plate was shaken at room temperature for 1 h, and 50 µL of stop solution was added to each well. Finally, the optical density was read at 405 nm using a Tecan reader. The average net optical density (OD) for each standard and sample was obtained by subtracting the average NSB density from the average OD for each sample and standard as follows

Average Net OD = Average OD − Average NSB OD(1)

For each pair of standard wells, the binding was calculated as a percentage of the maximum binding wells using the following formula:

(2) $$\mathrm{Percent}\;\mathrm{Bound}=\frac{\mathrm{Net}\;\mathrm{OD}}{{\mathrm{Net}\;B}_{0}\mathrm{OD}}\;\times \;100$$

For the standards, the calculated Percent Bound (B/Bo) was plotted vs. the concentration of serotonin. The concentration of serotonin in each sample was determined using this curve.

### 4.10. Statistical Analysis

Microsoft Excel 2019 was used for statistical analysis. The Student’s *t*-test was utilized. Statistical significance was expressed in terms of *p* values, with *p* < 0.05 considered to indicate statistical significance.

## 5. Conclusions

**Figure 7:**
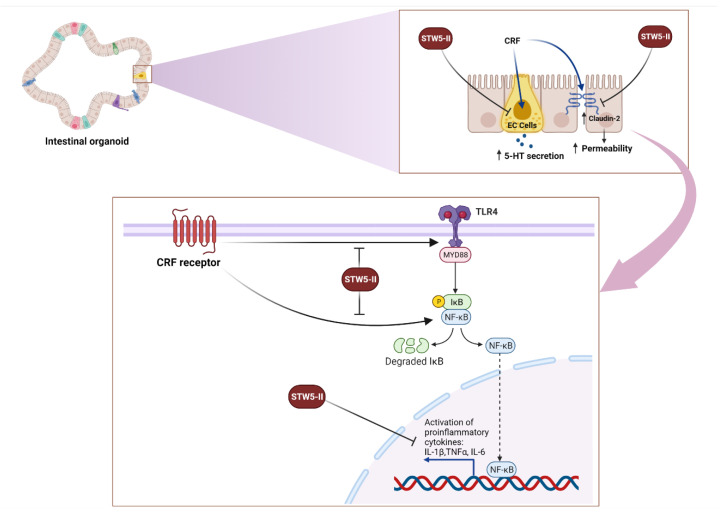
Multiple mechanistic activities of STW 5-II. STW 5-II suppresses CRF-induced TLR4 activation and its downstream regulator MYD88. STW5-II also inhibits NF-κB activation and the induction of proinflammatory cytokines. Finally, STW 5-II inhibits CRF-mediated upregulation of the tight junction protein Claudin-2. EC cells: Enterochromaffin cells, 5-HT: 5-hydroxytryptamine (Serotonin), IL1β: Interleukin1 beta, TLR4: Toll-like receptor 4, IL-6: Interleukin 6, TNFα: Tumor necrosis factor alpha, CRF: Corticotropin-releasing factor. Figure created with BioRender.com.

In conclusion, our results showed that STW 5-II effectively reduced CRF-mediated intestinal inflammation and cytokine secretion (Figure 7). It also inhibited the TLR4 system, which is a key player in CRF-mediated signaling, and restored the expression of *claudin-2*, thereby preserving the function of the intestinal barrier. Moreover, STW 5-II inhibited CRF-mediated serotonin secretion, which is an important mediator of IBS pathogenesis (Figure 7). To the best of our knowledge, this is the first work to demonstrate that CRF receptors are expressed in mouse intestinal organoids and to utilize these 3D models to recapitulate stress signaling and gut-brain interaction. Our results provide compelling evidence that STW 5-II could target different signaling pathways in the gut-brain axis, which explains its clinically proven efficacy for managing functional GI disorders, recently named DGBI. Further in vivo studies are needed to explore the detailed mechanisms by which STW 5-II alleviates stress-mediated IBS symptoms.

## Figures and Tables

**Table 1:** PyRx screening for top 10 compounds contained in STW 5-II with CRFR1.

Ligand	Binding Energy kcal/mol
Oleanolic acid	−8.9
Licorice glucoside C1	−8.9
Licorice saponin E2	−8.8
Yunganoside L2	−8.6
Glycyrrhetinic acid	−8.4
Rosmarinic acid dimer	−8.4
Glycyrrhizinic acid	−8.3
Licorice saponin G2	−8.3
Licorice saponin B2	−8.2
Licorice saponin B2 isomer	−8.2

**Table 2:** PyR screening for top 10 compounds contained in STW 5-II with CRFR2.

Ligand	Binding Energy kcal/mol
Licorice saponin G2	−9.2
Yunganoside L2	−9.2
Glycyrrhetinic acid	−9.1
Oleanolic acid	−9.0
Licorice saponin B2 isomer	−9.0
Licorice saponin_B2	−8.7
Rosmarinic acid dimer	−8.6
Cucurbitacin I	−8.5
Macedonoside-A-acetate	−8.5
Kaempferol-3,3″′,4′-tri-O-glucoside-7-O-rhamnoside	−8.5

**Table 3:** Docking results and binding interactions of oleanolic acid and licorice saponin G2 with CRFR1 and CRFR2, respectively.

Ligand	Target	Binding Energy kcal/mol	pKi (nM)	Amino Acids
Oleanolic acid	CRFR1	−8.6 ± 0.01	496.6 ± 9.1	LEU294, LEU213, TYR212, ALA315, LYS314, VAL318, TYR363, ARG151, HIS155
Licorice saponin G2	CRFR2	−10.07 ± ≤0.001	41.36 ± ≤0.001	LYS307, ARG306, LYS310, LEU313, VAL314, PRO317, PHE354, PHE358, PHE362

**Table 4:** List of qPCR primers.

Gene	Gene Symbol	Forward Primer	Reverse Primer
Glyceraldehyde-3-phosphate dehydrogenase	GAPDH	GAGGGATGCTGCCCTTACC	CAAATCCGTTCACACCGACC
Tumor necrosis factor-alpha	TNFα	TAGCCCACGTCGTAGCAAAC	ACAAGGTACAACCCATCGGC
Nuclear factor kappa B Subunit 1	NF-κB	CTCTGGCGAATGGCTTTACT	GAGGGGAAACAGATCGTCCA
Interleukin-1 beta	IL-1β	TGCCACCTTTTGACAGTGATG	AAAGGTTTGGAAGCAGCCCT
Interleukin-6	IL-6	ACCAAGAGATAAGCTGGAGTCA	TAACGCACTAGGTTTGCCGA
Claudin-2	CLDN2	ATGCCTTCTTGAGCCTGCTT	GCTGCTGCTCTTGCTTCTTG
Myeloid differentiation Primary response protein 88	MyD88	AGAGCTGCTGGCCTTGTTAG	GACTCCTGGTTCTGCTGCTT
Toll-like receptor 4	TLR4	TTCTTCTCCTGCCTGACACC	GTCATCAGGGACTTTGCTGAG

## Data Availability

Data is contained within the article.
